# Opposite feedback from mTORC1 to H-ras and K-ras4B downstream of SREBP1

**DOI:** 10.1038/s41598-017-09387-8

**Published:** 2017-08-21

**Authors:** Itziar M. D. Posada, Benoit Lectez, Farid A. Siddiqui, Christina Oetken-Lindholm, Mukund Sharma, Daniel Abankwa

**Affiliations:** Turku Centre for Biotechnology, Åbo Akademi University, Tykistökatu 6B, 20520 Turku, Finland

## Abstract

As a major growth factor transducer, Ras is an upstream activator of mTORC1, which further integrates nutrient and energy inputs. To ensure a contextual coupling of cell division via Ras/MAPK-signalling and growth via mTORC1-signalling, feedback loops from one pathway back to the other are required. Here we describe a novel feedback from mTORC1, which oppositely affects oncogenic H-ras- and K-ras-signalling output, and as a consequence stemness properties of tumourigenic cells. Amino acid stimulation of mTORC1 increases the processed form of SREBP1, a major lipidome regulator. We show that modulation of the SREBP1 levels downstream of S6K1 has opposite effects on oncogenic H-ras and K-ras nanoscale membrane organisation, ensuing signalling output and promotion of mammospheres expressing these oncogenes. Our data suggest that modulation of phosphatidic acid, a major target of SREBP1 controlled lipid metabolism, is sufficient to affect H-ras and K-ras oppositely in the membrane. Thus mTORC1 activation increases H-ras-, but decreases K-ras-signalling output in cells transformed with the respective oncogene. Given the different impact of these two Ras isoforms on stemness, our results could have implications for stem cell biology and inhibition of cancer stem cells.

## Introduction

The mechanistic target of rapamycin (mTOR) is tightly associated with several metabolic diseases and cancer^1^. Two major complexes built around the mTOR-kinase are distinguished, mTOR complex 1 (mTORC1; characterized by Raptor) and mTORC2 (with Rictor as major constituent). For historical reasons, the rapamycin-inhibited mTORC1 is better characterized than mTORC2 in its activities. While both are responsive to growth factor stimulation, only mTORC1 is known as a major integrator of essential metabolites, with both ATP and amino acids stimulating the pathway^2^. Thus mTORC1 is central to driving anabolic cellular processes (e.g. protein and lipid synthesis), while blocking catabolism (e.g. autophagy).

Given this broad cell physiological involvement, activation of mTORC1 proceeds via various signalling pathways. The canonical activation downstream of Ras involves inhibition of the heterodimeric tuberous sclerosis complex 1/2 (TSC1/2) by Erk and Akt. As the TSC1/2 complex negatively regulates the small GTPase Ras homolog enriched in brain (Rheb) due to its GTPase-activating protein (GAP) activity, growth factor stimulation supports interaction between GTP-Rheb and mTORC1. In addition, mTORC1 needs to be recruited onto cellular organelles that are decorated by Rheb.

At least three distinct amino acid dependent sensing systems enable this. The first one depends on heterodimers of Rag GTPases and the Ragulator complex, which recruit mTORC1 to the lysosome^3, 4^. The second one requires ADP ribosylation factor-1 (Arf1) and vacuolar H^+^-adenosine triphosphatase (v-ATPase) also on the lysosome and responds to glutamine, not leucine as the former^5^. A third amino acid sensing machinery operates from the Golgi apparatus, where GTP-bound Rab1a is required for mTORC1 engagement by Rheb^6^.

The activated mTORC1 complex has well characterized effectors that are linked to major cellular homeostatic functions, such as the control of protein levels. Both the translation factor eIF4E-binding protein 1 (4E-BP1) and ribosomal protein S6 (S6) kinase 1 (S6K1) are directly phosphorylated by mTORC1^1^. More recently mTORC1 was also connected to regulating proteasome-mediated protein degradation^7^.

S6K1 is furthermore a major positive regulator of the master transcription factors for lipogenesis, the sterol regulatory element binding proteins (SREBP) 1 and 2^8^. While the complex mechanism of SREBP activation is still being investigated, it is known that it involves proteolytic processing and nuclear translocation^8^. Importantly, both SREBP1 and 2 promote proliferation^9^, however, the mechanistic basis for this is not fully understood. It is plausible that increased expression of transcriptional targets of SREBP1/2 that provide lipids as the building blocks for cellular growth is one necessary element to sustain proliferation^10^. Consistently, targets downstream of SREBP, such as FASN (fatty acid synthase) are elevated in cancer and established drug targets^11^.

In agreement with the central role of the mTOR-pathway a multitude of complex feedback and crosstalk mechanism exist that harness basically all means of protein activity regulation, including translational control, phosphorylation and degradation^12^. Notably, inhibition of mTORC1 by rapamycin can activate the phosphatidylinositol 3-kinase (PI3K) pathway, via a negative feedback loop from S6K1 via IRS-1 (insulin receptor substrate-1) to PI3K^13^. An extension of this feedback from PI3K to Ras furthermore re-activates the mitogen-activated protein kinase (MAPK) signalling in response to rapalog treatment^14^. However, feedback-control by the altered metabolite landscape of the cell is less well characterized.

As the master relay switch of mitogenic stimuli, Ras is central not only to MAPK-signalling, but also an upstream input of mTORC1. The Ras effector PI3K initiates activation of Akt, one of the major upstream regulators of mTORC1-signalling^15^. The three RAS genes HRAS, NRAS and KRAS display increasing frequencies of activating mutations in cancer in this order^16^. While the exact reasons for this are still poorly understood, the high frequency of KRAS mutations have led to its nomination as the primary Ras drug-target^17^. Biochemically, Ras proteins are almost identical in their properties, as they all promiscuously activate several effector pathways^18^. However, when examining their role during development, it becomes apparent that Ras proteins must have distinct functions. This is not least supported by the different knock-out phenotypes, where only KRAS deletions are embryonically lethal^19–23^. Moreover, the distinct roles of the two KRAS splice isoforms K-ras4A and K-ras4B are insufficiently understood^22, 24^. Additional data suggest that K-ras and H-ras in particular can adopt opposite functions, as only K-ras4B drives self-renewal in stem cells, while H-ras promotes differentiation and N-ras being apparently neutral^25^. In line with this, K-ras4B (hereafter K-ras) is an exquisite driver of cancer stem cells (CSC) and the common mechanistic target of a number of CSC-drugs^26, 27^.

These opposite and divergent functions in particular of H-ras and K-ras emerge within the plasma membrane, such as by various protein assisted mechanisms^28, 29^. Intriguingly, oncogenic H-ras can itself negatively regulate the nanoscale organisation of K-ras in the plasma membrane (nanoclustering) by perturbing the phosphatidylserine (PS) distribution^30^. In line with this, segregation of PS by caveolae can downmodulate K-ras nanoclustering^31^. A number of other acidic lipids, including phosphatidic acid (PA) and phosphatidylinositol (4,5)-bisphosphate (PIP_2_) characterise Ras nanodomains^30, 32^.

Importantly, dynamic nanoclustering of Ras into distinct di- or oligomeric signalling complexes, correlates with effector recruitment and MAPK-signalling output^33, 34^. Hence, nanoclustering can be modulated to alter Ras-signalling output, such as by modifying membrane lipid organisation^30, 35^ or changing the abundance of nanocluster scaffolding/modulator proteins, such as galectin-1 or apoptosis-stimulating of p53 protein 2 (ASPP2)^33, 36^.

Here, we describe how activation of the mTORC1-pathway by increasing amino acid levels can specifically stimulate stemness properties of oncogenic H-ras expressing cells, while repressing stemness in cells expressing oncogenic K-ras. This is a consequence of an unrecognized feedback from the mTORC1-pathway downstream of SREBP1 to the nanoscale membrane organisation of Ras. We show that increasing levels of SREBP1, relatively downmodulate PA levels. Intriguingly, this has opposite effects on H-ras- and K-ras-nanoclustering, thus impacting on MAPK-signalling output and mammosphere growth.

## Results

### Structurally diverse protein synthesis inhibitors inversely regulate oncogenic H-ras- and K-ras-nanoclustering

Recent data suggest that Ras isoforms H-ras and K-ras4B (hereafter K-ras) have partially opposing functions in stem cells and cancer stem cells^25–27^, indicating that Ras isoform-specific inhibitors might be required in cancer therapy. We therefore followed up on our recent findings, showing that the protein synthesis inhibitor cycloheximide (CHX) specifically increases H-ras nanoclustering (i.e. nanoscale membrane signalling complexes of Ras^37^) and downstream signalling, whereby it paradoxically promoted mammosphere growth in a H-ras-dependent manner^38^.

**Figure 1:**
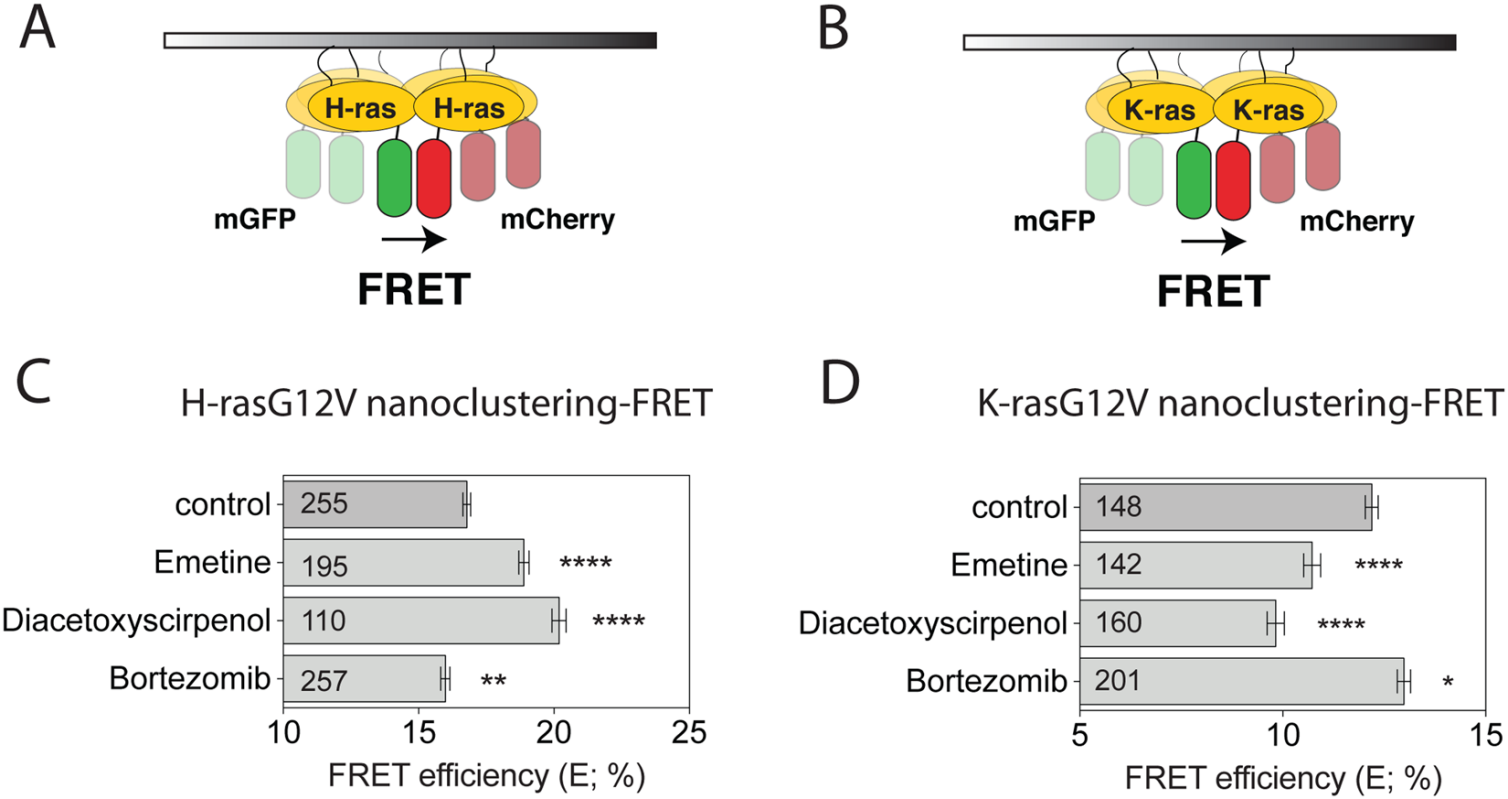
Protein synthesis inhibitors inversely regulate oncogenic H-ras and K-ras nanoclustering. (**A**,**B**) Schematic illustration of the nanoclustering-FRET assay. FRET is increased due to the formation of transiently immobile signalling complexes, which lead to nanoscale clustering of H-ras (**A**) or K-ras (**B**) in the plasma membrane. (**C**,**D**) Nanoclustering-FRET analysis in HEK cells co-expressing mGFP- and mCherry-tagged (**C**) H-rasG12V or (**D**) K-rasG12V. Cells were treated for 24 h with DMSO control, or 2 μM of the protein synthesis inhibitors diacetoxyscirpenol and emetine, or 0.4 μM of the proteasome inhibitor bortezomib. The numbers in the bars indicate the number of analyzed cells (mean ± SEM, n = 3). Statistical significance levels are annotated as *p < 0.05; **p < 0.01; ****p < 0.0001.

In order to monitor Ras nanoclustering changes, we used our well established Ras nanoclustering-FRET assay in HEK293EBNA cells (hereafter HEK)^28, 36^. In this assay, we genetically fuse FRET-pairs mGFP and mCherry to the free N-terminus of Ras and monitor the emergence of FRET, due to the tight packing in nanoclusters (Fig. 1A,B). Loss of FRET can report on either disintegration of membrane nanocluster or loss of membrane attachment, while increased FRET is only consistent with increased nanoclustering of Ras^27^. Interestingly, structurally diverse protein synthesis inhibitors (PSI) increased H-ras nanoclustering-FRET (Fig. 1C). However, in contrast to CHX they decreased nanoclustering-FRET of K-ras (Fig. 1D and Supplementary Fig. 1A) without altering K-ras subcellular localization (Supplementary Fig. 1B). Unlike CHX^38^, the other PSI robustly blocked mammosphere formation of MDA-MB-231 cells, as would be expected from the inhibition of cellular protein synthesis (Supplementary Fig. 1C).

In order to understand these different observations, we wondered whether other attendant properties of PSI could be responsible for some of the observed CHX effects. A common denominator of all of these PSI, is their ability to increase intracellular amino acid levels (Supplementary Fig. 1D). In agreement with amino acid levels influencing the nanoclustering response, lowering the amount of amino acids with the proteasome inhibitor bortezomib (Supplementary Fig. 1D) decreased H-ras nanoclustering-FRET (Fig. 1C), while increasing that of K-ras (Fig. 1D). However, bortezomib and another proteasome inhibitor MG132 robustly blocked mammosphere growth of K-ras mutant MDA-MB-231 cells (Supplementary Fig. 1C), in line with the requirement of proteasome activity for cellular homeostasis and stemness^39, 40^.

### Increased amino acid levels oppositely affect signalling output from the two Ras isoforms

**Figure 2:**
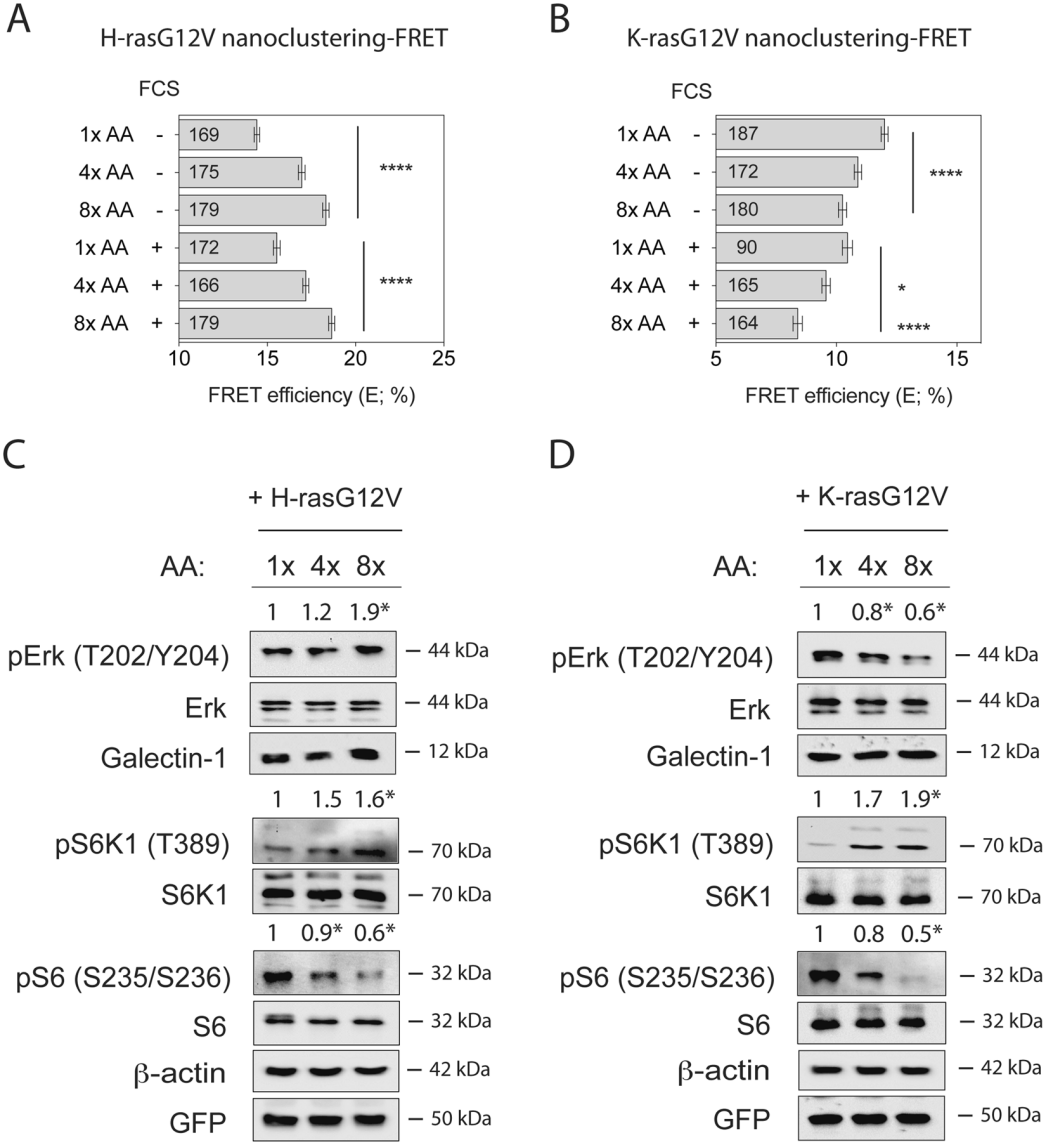
Amino acid increase oppositely affects H-ras and K-ras activity. (**A**,**B**) Nanoclustering-FRET analysis in HEK cells co-expressing mGFP- and mCherry-tagged (**A**) H-rasG12V or (**B**) K-rasG12V. Cells were incubated for 24 h with increasing concentrations of amino acids (1x, 4x or 8x) in the absence or presence of serum (FCS) as indicated. The numbers in the bars indicate the number of analyzed cells (mean ± SEM, n = 3). (**C**,**D**) Western blot analysis of Ras- and mTORC1-signalling in HEK cells expressing mGFP-H-rasG12V or mGFP-K-rasG12V. Cells were treated as in (**A**,**B**) under serum-starved conditions. Numbers indicate the ratio of phosphorylated to respective total protein levels (n = 4). Statistical significance levels are annotated as *p < 0.05; ****p < 0.0001.

These data suggested that modulation of the intracellular amino acid levels oppositely affect H-rasG12V and K-rasG12V nanoclustering, while protein synthesis and proteasome activity would have to remain intact for these changes to affect mammosphere growth. We therefore next examined whether direct modulation of the cellular amino acid levels would change Ras nanoclustering and downstream signalling. Indeed, increasing amino acid levels increased H-ras nanoclustering-FRET in the same manner with and without serum (Fig. 2A). At relatively lowered amino acid levels (1x AA; 4x AA corresponds to normal), a clear H-ras mislocalization from the plasma membrane to the cytosol could be observed (Supplementary Fig. 2A). By contrast, K-ras nanoclustering-FRET decreased with increasing levels of amino acids, even more so in the presence of serum (Fig. 2B). Nevertheless K-ras plasma membrane localization remained unaltered (Supplementary Fig. 2B).

MAPK-signalling follows changes in Ras nanoclustering, due to the fact that Raf effectors are only recruited to nanoclustered Ras^33, 34^. In agreement with this, increasing amounts of amino acids under serum starvation significantly increased phospho-T202/Y204-Erk (pErk) levels in H-rasG12V transfected HEK cells (Fig. 2C), but significantly decreased pErk levels in K-rasG12V transfected HEK (Fig. 2D).

We recently showed that the small lectin galectin-1 (Gal-1) increases oncogenic H-ras- but decreases K-ras-nanoclustering to a similar extent as observed here after amino acid level increase^28, 29^. However, expression of Gal-1 remained unaltered with increasing amino acid concentrations in both H-rasG12V and K-rasG12V expressing HEK cells (Fig. 2C,D), suggesting that it is not responsible for the observed changes in nanoclustering.

Given that amino acid abundance is sensed by mTORC1-signalling^1^, we also examined its downstream activity. In variation to the pErk-response pattern, phospho-T389-S6K1 (pS6K1) levels increased significantly in both of the Ras transfected or wild type (wt) HEK cells, while surprisingly phospho-S235/S236-S6 (pS6) levels decreased with increasing amino acid levels (Fig. 2C,D and Supplementary Fig. 2C). This may represent an unknown feedback response from protein translation, where the overabundance of amino acids is countered by reduced levels of pS6 in order to prevent proteotoxic stress.

### Tumourosphere growth follows Ras nanoclustering and MAPK-signalling

Given that amino acid stimulation robustly increased mTORC1-signalling, we investigated, whether this would support mammosphere growth. The number of mammospheres reports on the population of CD44^+^/CD24^−^ mammary stem cells, which can grow under low serum and non-adherent conditions. Cancer cells enriched for these properties have cancer stem cell properties, i.e. they very efficiently induce tumours^41^.

**Figure 3:**
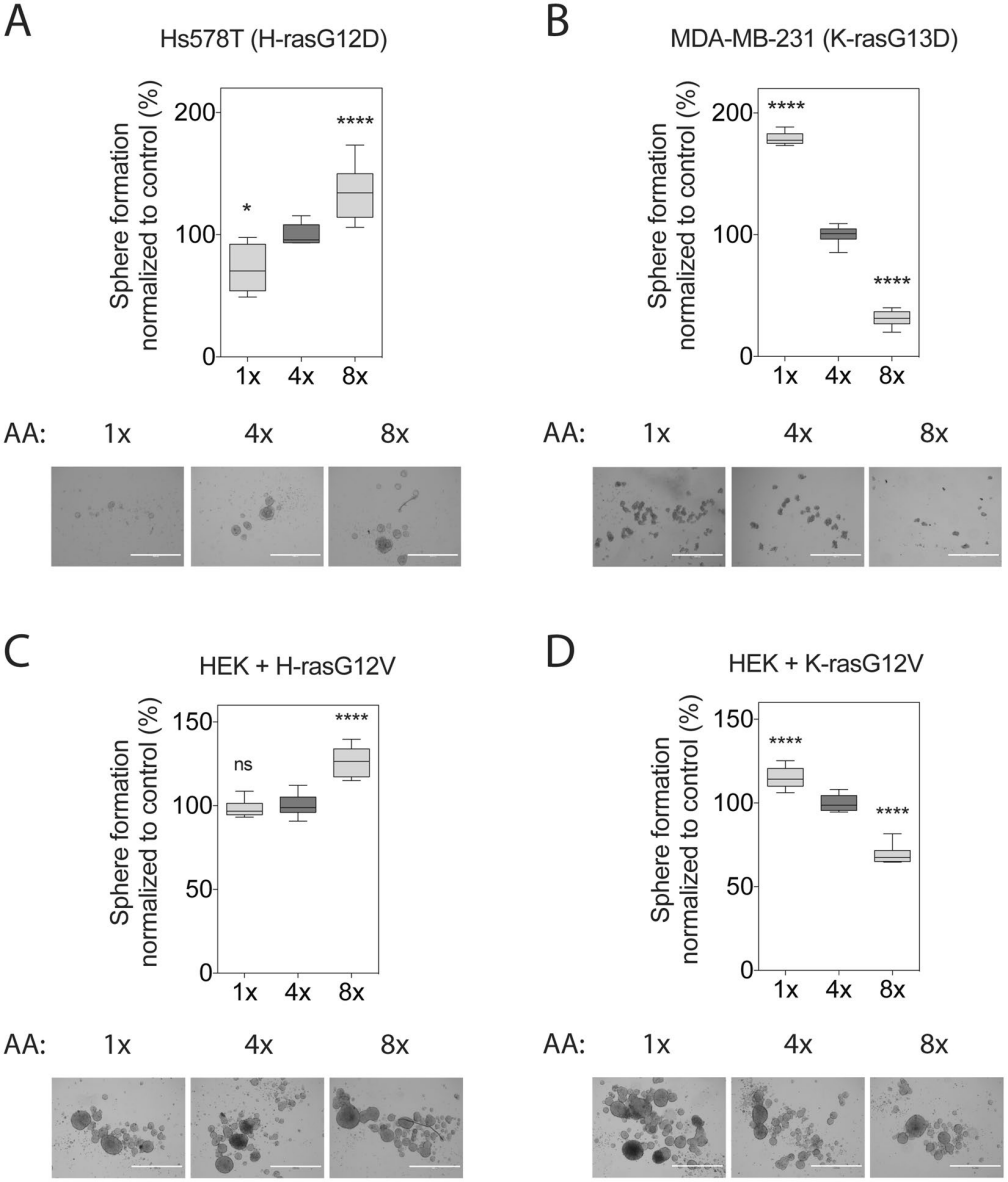
Tumourosphere growth follows Ras nanoclustering and MAPK-signalling. Sphere-forming efficiency of the breast cancer cell lines (**A**) Hs578T or (**B**) MDA-MB-231, and of the oncogenic (**C**) H-ras- or (**D**) K-ras-expressing HEK cells, grown under increasing amino acid concentrations for 9 days (n = 3). Representative images of spheres are shown below the plots. Scale bar in the images represents 1000 μm. Statistical significance levels are annotated as ns, not significant; *p < 0.05; ****p < 0.0001.

Intriguingly, mammosphere growth was qualitatively tuned exactly as the amino acid induced Ras nanoclustering and MAPK-signalling response. H-rasG12D mutated Hs578T cells increased their sphere-forming capacity with increasing amounts of amino acids (Fig. 3A). By contrast, K-rasG13D mutated MDA-MB-231 cell derived sphere numbers decreased dramatically with increasing levels of amino acids (Fig. 3B).

The breast cancer cell lines Hs578T and MDA-MB-231 feature several oncogenic mutations, in the MAPK- and PI3K-pathway. In order to validate that the sphere growth response was indeed mainly guided by the expressed oncogenic Ras isoform, we employed a HEK-sphere model, which is based on a breast cancer stem cell marker positive and tumourigenic population in the common HEK cell line^42^. In agreement with the mammosphere data, HEK-sphere numbers were increased in both the H-rasG12V background (Fig. 3C) as well as the wt background (Supplementary Fig. 3), but decreased in the K-rasG12V background when amino acid levels were raised (Fig. 3D).

Therefore, we conclude that increasing the intracellular amino acid level does not only stimulate mTORC1-signalling, but also promotes H-ras nanoclustering, MAPK-signalling and mammosphere formation, unless cells express oncogenic K-ras, in which case the inverse is observed.

### Ablation of mTORC1-pathway components down to S6K1 affects only H-ras-nanoclustering and -signalling

**Figure 4:**
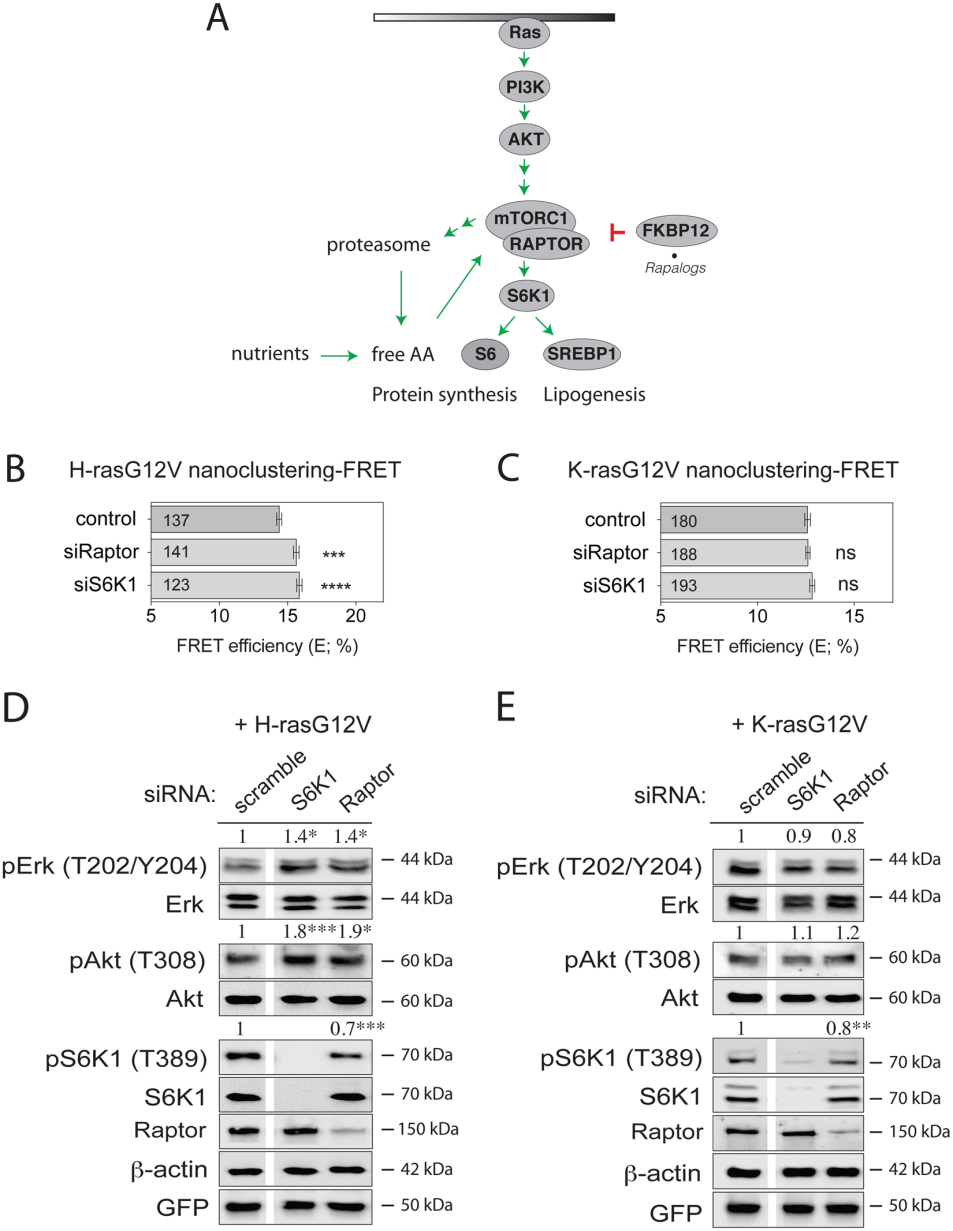
The ablation of mTORC1 pathway components Raptor and S6K1 increases specifically H-ras activity. (**A**) Schematic illustration of the basic mTORC1-signalling pathway and its relation to Ras output. We focused on proteins, whose expression levels were manipulated in this study. (**B**,**C**) Nanoclustering-FRET analysis in HEK cells transfected with scrambled siRNA or siRNA directed to Raptor or S6K1, and co-expressing mGFP- and mCherry-tagged (**B**) H-rasG12V or (**C**) K-rasG12V. The numbers in the bars indicate the number of analyzed cells (mean ± SEM, n = 4). (**D**,**E**) Western blot analysis of Ras- and mTORC1-signalling in HEK cells transfected with the indicated siRNA, and expressing (**D**) mGFP-H-rasG12V or (**E**) mGFP-K-rasG12V. Numbers indicate the ratio of phosphorylated to respective total protein levels (n = 3). Statistical significance levels are annotated as ns, not significant; *p < 0.05; **p < 0.01; ***p < 0.001; ****p < 0.0001.

We hypothesized that Ras-signalling stimulation is the result of a feedback from mTORC1-signalling. In order to understand at which level of the mTORC1-pathway the inverse effects on H-ras- and K-ras-nanoclustering set in, we genetically dissected the pathway in HEK cells using siRNA-mediated knockdown (Fig. 4A).

Knockdown of mTORC1 specific Raptor or of S6K1 increased only H-ras nanoclustering-FRET (Fig. 4B,D), but did not affect K-ras nanoclustering-FRET (Fig. 4C,E). Consistently, pErk and phospho-T308-Akt (pAkt) levels were significantly increased in H-rasG12V expressing cells (Fig. 4D), while they were not significantly changed in those expressing K-rasG12V (Fig. 4E).

### Increased levels of mature SREBP1 inversely regulate signalling output and sphere growth driven by H-ras or K-ras

**Figure 5:**
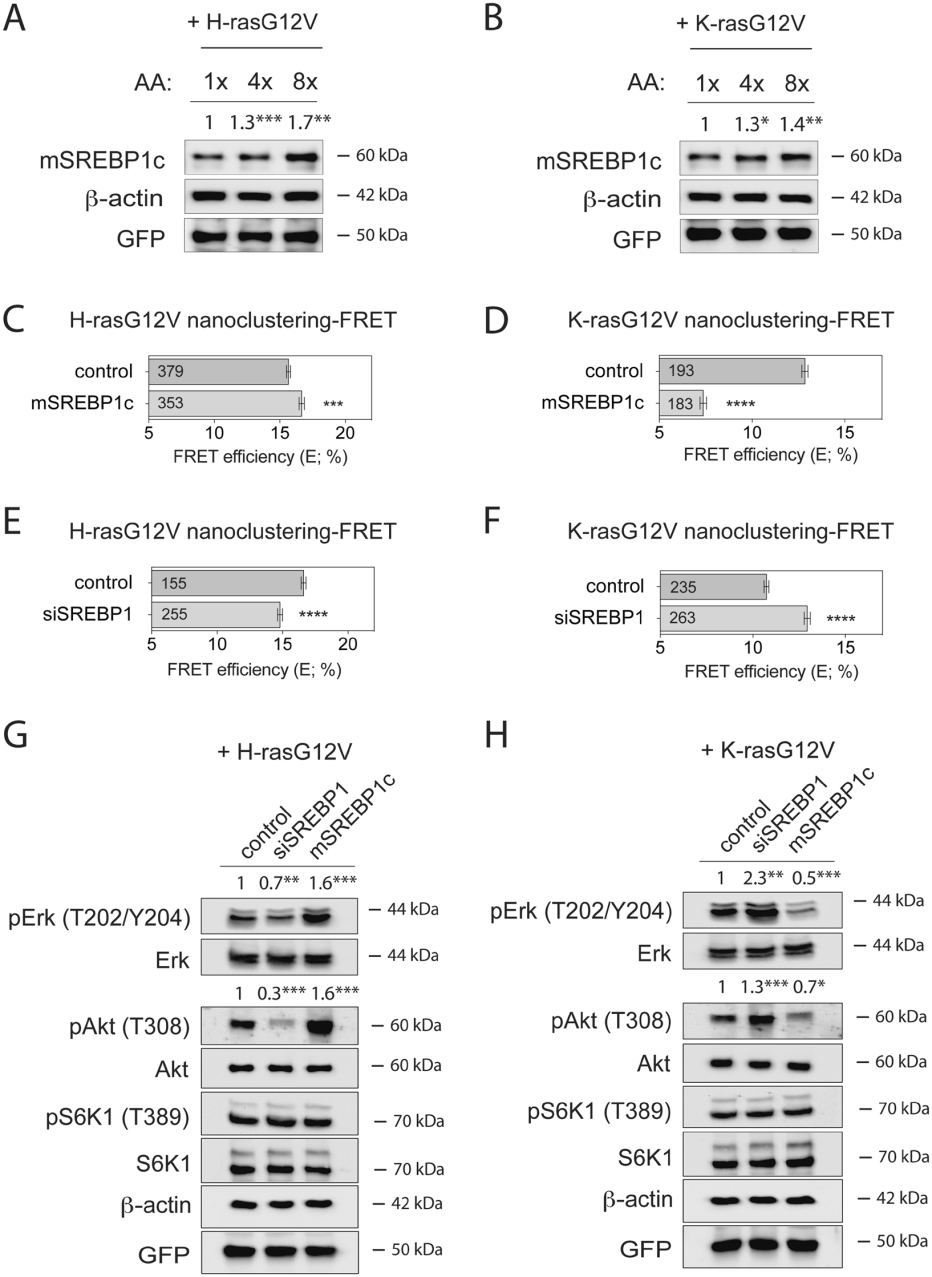
mSREBP1c inversely regulate signalling output driven by oncogenic H-ras or K-ras. (**A**,**B**) Western blot analysis of mSREBP1c in HEK cells transiently expressing (**A**) mGFP-H-rasG12V or (**B**) mGFP-K-rasG12V. Cells were incubated for 24 h with increasing concentrations of amino acids (1x, 4x or 8x) in the absence of serum. Numbers indicate β-actin normalized mSREBP1 levels (n = 3). (**C**,**D**) Nanoclustering-FRET analysis in HEK cells transfected with mature Flag-tagged mSREBP1c or empty vector, co-expressing mGFP- and mCherry-tagged (**C**) H-rasG12V or (**D**) K-rasG12V. The numbers in the bars indicate the number of analyzed cells (mean ± SEM, n = 4). (**E**,**F**) Nanoclustering-FRET analysis in HEK cells transfected with scrambled siRNA or siRNA directed to SREBP1, and co-expressing mGFP- and mCherry-tagged (**E**) H-rasG12V or (**F**) K-rasG12V. The numbers in the bars indicate the number of analyzed cells (mean ± SEM, n = 4). Note that the variations in FRET efficiency (%) of the control levels in C, E and D, F are due to the different experimental procedures. (**G**,**H**) Western blot analysis of Ras- and mTORC1-signalling in HEK cells transfected with siRNA SREBP1 or Flag-tagged mSREBP1c vector, transiently expressing (**G**) mGFP-H-rasG12V or (**H**) mGFP-K-rasG12V. Numbers indicate the ratio of phosphorylated to respective total protein levels (n = 3). Statistical significance levels are annotated as *p < 0.05; **p < 0.01; ***p < 0.001; ****p < 0.0001.

Another prominent target of S6K1 is SREBP1, which is known as a master lipidome regulator^9^. Consistent with the activation of S6K1 after amino acid stimulation (Fig. 2C,D and Supplementary Fig. 2C), we found that amino acid abundance also regulated the abundance of the mature and active form of SREBP1 (mSREBP1c) in HEK cells (Fig. 5A,B and Supplementary Fig. 4A). In support of amino acids oppositely regulating H-ras and K-ras by modulating mature and active SREBP1 levels downstream of S6K1, we found that the constitutively active mutant of S6K1 (caS6K1) and mSREBP1c positively regulated H-ras nanoclustering-FRET, while negatively K-ras nanoclustering-FRET (Fig. 5C,D and Supplementary Fig. 4B,C). Hence, these manipulations both mimicked the effect of amino acid stimulation (Fig. 2). Correspondingly, the inverse was true if SREBP1 was downmodulated (Fig. 5E,F and Supplementary Fig. 4D).

In support of the link between the effect of amino acids and SREBP1 activity, siSREBP1 treatment was accompanied by a weak redistribution of H-ras to the cytoplasm (Supplementary Fig. 4E), similar to what was observed with reduced amino acid content (1xAA) (Supplementary Fig. 2A). Similarly, mSREBP1c expression strongly decreased K-ras-FRET, but did not visibly affect its distribution (Supplementary Fig. 4F), consistent with our observations with increased amino acid levels (Supplementary Fig. 2B).

Moreover, these modulations of SREBP1 activity altered not only pErk, but also pAkt levels in correlation with the nanoclustering-FRET data (Fig. 5G,H). Importantly, pS6K1 levels remained unchanged (Fig. 5G,H), suggesting that nanoclustering and signalling changes emerged from a feedback downstream of SREBP1 to Ras nanoclustering and not from effects on mTORC1-signalling.

### H-ras- and K-ras-driven stemness properties are oppositely altered downstream of SREBP1

**Figure 6:**
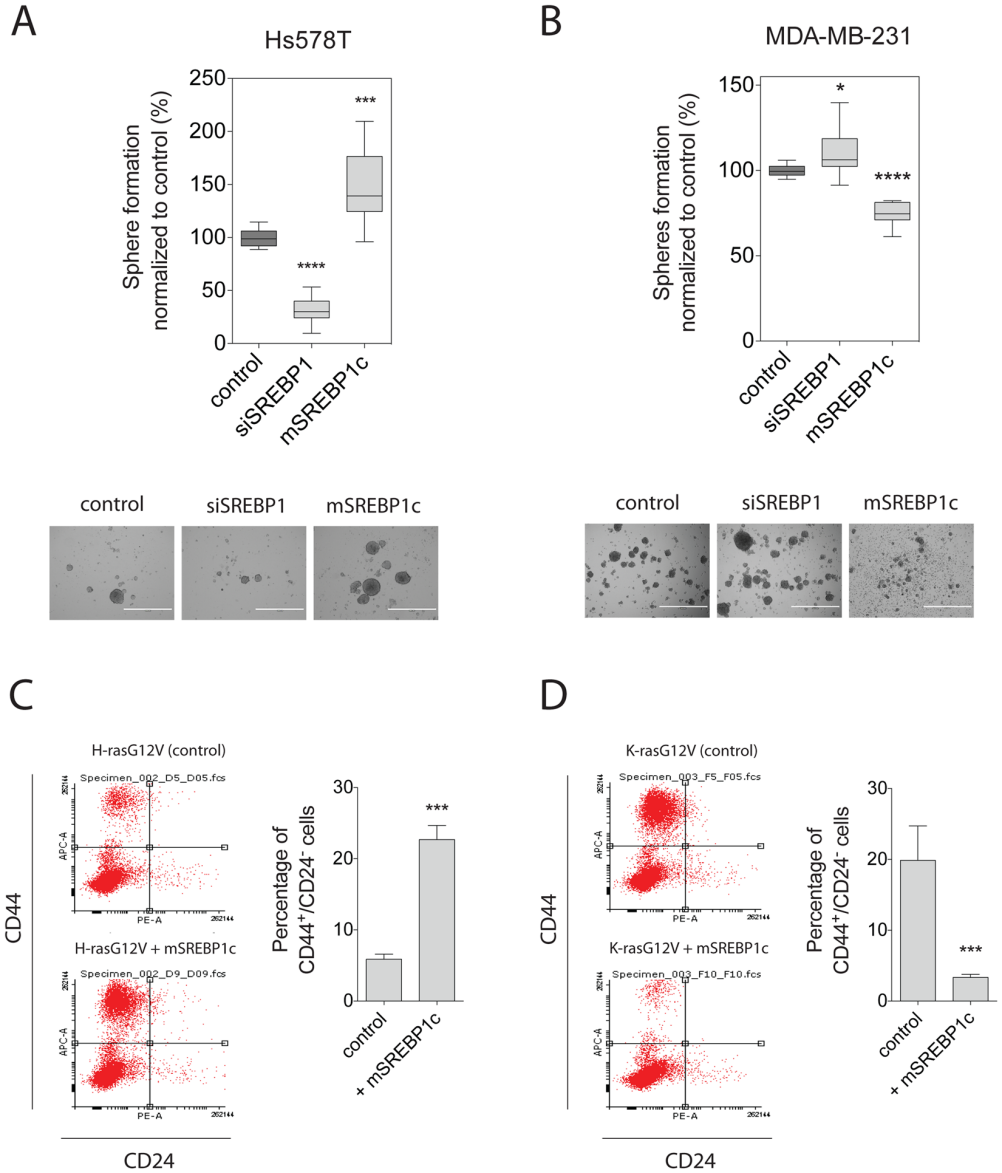
Ras isoform-driven stemness properties are oppositely altered by mSREBP1c. (**A**,**B**) Mammosphere-forming efficiency was measured for Hs578T (**A**) or MDA-MB-231 (**B**) breast cancer cells transfected with SREBP1 siRNA or mSREBP1c vector. Mammospheres were allowed to form for 9 days. The mammosphere-forming efficiency was measured as the number of spheres formed and normalized to control (mean ± SEM, n = 3). Representative images of spheres are shown below the plots. Scale bar represents 1000 μm. (**C**,**D**) *Left*, CD44/CD24 FACS profiles are shown for HEK cells expressing (**C**) H-rasG12V or (**D**) K-rasG12V and after mSREBP1c expression. *Right*, shown is the average percentage of CD44^+^/CD24^−^ HEK cells for each condition. Error bars denote the SEM from three independent experiments performed in duplicate. Statistical significance levels are annotated as *p < 0.05; ***p < 0.001; ****p < 0.0001.

In order to test, whether the opposite effect on tumourosphere formation by amino acid stimulation of the mTORC1 pathway (Figs 2 and 3) originated downstream of SREBP1, we analysed the direct effect of SREBP1 modulation on mammosphere growth. Consistent with the effect on H-ras nanoclustering-FRET and signalling (Fig. 5C,E,G), knockdown of SREBP1 significantly decreased sphere formation, while expression of mSREBP1c increased sphere formation of H-ras mutated Hs578T (Fig. 6A). As expected from the K-ras data (Fig. 5D,F,H), the opposite was seen in K-ras mutated MDA-MB-231 cells (Fig. 6B).

We next analysed additional stemness properties using our HEK-sphere model again. In this model, H-rasG12V can induce a CD44^+^/CD24^−^ stem-like population (Fig. 6C and Supplementary Fig. 5A), however to a lesser extent than K-rasG12V (Supplementary Fig. 5B). In agreement with the mammosphere increasing effect of mSREBP1c in oncogenic H-ras transformed breast cancer cells (Fig. 6A), expression of mSREBP1 in H-rasG12V-HEK-spheres increased the stem-like population significantly. Conversely, expression of mSREBP1c in K-rasG12V HEK-spheres significantly decreased CD44^+^/CD24^−^ stem-like population (Fig. 6D), consistent with what was observed in MDA-MB-231 cells expressing mSREBP1c (Fig. 6B).

These data suggest that downstream of SREBP1 a feedback mechanism to Ras exists, which oppositely affects H-ras- and K-ras-associated nanoclustering, signalling and stemness properties in oncogenic Ras-expressing cells.

### Downstream of SREBP1 altered phosphatidic acid levels inversely affect H-ras and K-ras nanoscale membrane organization

In order to understand, how an opposite effect for the two Ras isoforms could result from a unidirectional change of mSREBP1c (Fig. 5A,B), we considered the lipidomic effects of SREBP1. Knockdown of SREBP1 downregulates the phosphatidate phosphatase lipin 1, which converts phosphatidic acid (PA) to diacylglycerol^43^. Thus knockdown of SREBP1 or inhibition of lipin 1 leads predominantly to a net accumulation of PA, while concomitantly other lipids that use PA as a precursor, such as phosphatidylserine, are lost^10^. Recent data by the Hancock group suggested specific effects in particular of acidic lipids on H-ras and K-ras nanoscale membrane organization^30, 44^.

**Figure 7:**
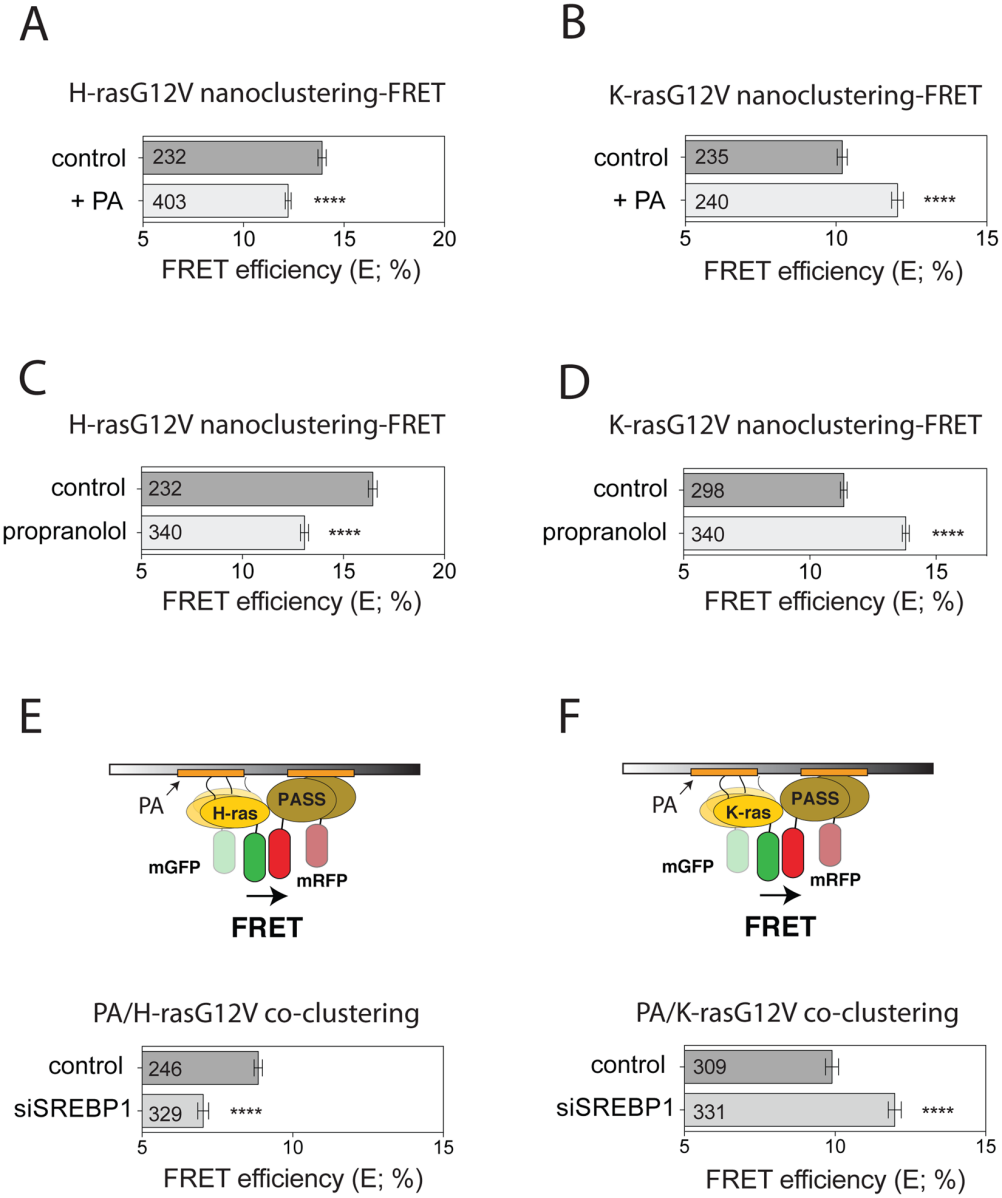
Altered phosphatidic acid levels inversely affect H-ras and K-ras nanoclustering-FRET. (**A**,**B**) Nanoclustering-FRET analysis in HEK cells expressing mGFP- and mCherry-tagged (**A**) H-rasG12V or (**B**) K-rasG12V. Cells were incubated for 45 min with externally added 100 μM phosphatidic acid (PA). The numbers in the bars indicate the number of analyzed cells (mean ± SEM, n = 3). (**C**,**D**) Nanoclustering-FRET analysis in HEK cells expressing mGFP- and mCherry-tagged (**C**) H-rasG12V or (**D**) K-rasG12V. Cells were treated for 30 min with DMSO control or 100 μM of propranolol. The numbers in the bars indicate the number of analyzed cells (mean ± SEM, n = 3). (**E**,**F**) Co-clustering FRET analysis (illustrated in schemes) using FLIM in HEK cells transfected with scrambled or siRNA directed to SREBP1, expressing (**E**) mGFP-H-rasG12V or (**F**) mGFP-K-rasG12V and mRFP-PASS probe. The numbers in the bars indicate the number of analyzed cells (mean ± SEM, n = 3). Statistical significance levels are annotated as ****p < 0.0001.

Consistent with PA affecting the nanoscale membrane organisation of Ras proteins, addition of PA to HEK cells was sufficient to decrease nanoclustering-FRET of H-rasG12V (Fig. 7A), thus mimicking the effect of the SREBP1 knockdown (Fig. 5E). Intriguingly, the opposite effect by this treatment was observed on K-rasG12V (Fig. 7B). In agreement with a SREBP1 - lipin 1 -axis dependence, inhibition of lipin 1 with propranolol, which should increase PA levels, also phenocopied these nanoclustering-FRET effects (Fig. 7C,D).

In support of PA-levels affecting directly the nanoscale membrane organisation of the two Ras isoforms in an opposite manner, knockdown of SREBP1 decreased co-clustering-FRET of a PA-specific probe (PASS)^45^ with H-rasG12V (Fig. 7E), while increasing that with K-rasG12V (Fig. 7F). Thus modulation of PA levels is sufficient to inversely regulate H-ras- and K-ras-nanoscale membrane organisation.

## Discussion

Given that the mTORC1-pathway is central to cellular growth regulation, it is not surprising that it is spiked with multiple feedback and feedforward mechanisms^12^. Canonical Ras-signalling stimulates via its effector PI3K and Akt directly the mTORC1-pathway. Thus oncogenic K-ras and PI3K can induce de novo lipid synthesis via SREBP1/2^46^. Correspondingly, feedback mechanisms from mTORC1 back to PI3K- and Ras/MAPK-signalling are known^13^. While it was established before that the MAPK-feedback is PI3K-dependent^14^, exact details on how this is effected remained unresolved.

Our results suggest two novel types of feedback responses to H-ras- and K-ras-nano/microscale membrane organisation and signalling from the mTORC1-pathway. Firstly, disruption of the mTORC1-pathway down to the level of S6K1 specifically increases H-ras nanoclustering, as well as pErk and pAkt only in cells expressing oncogenic H-ras (Fig. 4). However, we do not further examine the mechanism for this type of feedback here. Secondly, downstream of SREBP1, mTORC1 output from S6K1 increases H-ras-, but decreases K-ras-nanoclustering-FRET and modulates MAPK-signalling correspondingly by a mechanism that depends on the lipidome remodelling activity of SREBP1. This latter opposite feedback has important consequences for the promotion of stemness properties in tumourigenic cells (Fig. 6). Others and we observed that cells expressing oncogenic H-ras have a significantly lower potential to induce tumourospheres and stemness markers than those expressing oncogenic K-ras^26^ (Supplementary Fig. 5B). However, upon amino acid mediated stimulation of mTORC1, stemness properties of oncogenic H-ras expressing cells are increased, while surprisingly those of oncogenic K-ras expressing cells are dramatically decreased (Fig. 3). We explain this by the increased activity of SREBP1 downstream of mTORC1, which would relatively suppress PA levels^10^. Importantly, H-ras and K-ras nanoscale clustering is oppositely affected by PA levels, leading to the observed opposite effects in downstream signalling and promotion of stemness properties that depend on the oncogenic Ras isoform (Fig. 7).

Alternatively, changes in the levels of other lipids that are regulated by SREBP1 contribute to our observations. Notably, SREBP1 knockdown also decreases PS levels^10^. PS is indeed another critical player that maintains the correct nanoscale membrane organisation of H-ras and K-ras^30, 44, 47^. A specific level of PS is required to maintain lateral segregation of K-ras from H-ras. Moreover, only nanocluster of active K-ras, but not H-ras depends on PS. Yet, oncogenic H-ras can compete for PS, thus remotely decreasing K-ras nanocluster and signal output^30^. However, we conclude that the PA effect is dominant in our case, as the PS-associated changes (loss of PS decreases active K-ras nanocluster and does not affect H-ras nanocluster) would be inconsistent with our observations.

In support of the particular relevance of PA, its role to promote C-Raf membrane translocation may be significant^48^. As a consequence of increased PA the concentration of Raf-effectors on the plasma membrane would increase, which could facilitate their dimerization. Dimerization of Raf is not only necessary for Raf activation^49^, but also associated with the differential promotion of K-ras- and H-ras-nanoclustering^50, 51^. This could represent a mechanism for how increased PA levels would positively regulate K-ras nanocluster.

SREBP1/2 are furthermore known to modulate the mevalonate pathway, which provides cholesterol and prenyl-diphosphates^52^. Cholesterol is required for proper H-ras membrane organisation^53^, and together with PA and PS may be required to maintain proper lateral segregation of Ras proteins^32^. It can be assumed that correct lateral segregation of Ras proteins into specific proteo-lipid domains provides selective engagement with certain Ras effectors, similar to what was just described for Raf, and is therefore necessary for Ras isoform specific signalling output.

Also prenyl-diphosphates downstream of the SREBP1/2-controlled mevalonate pathway are relevant for Ras. They are required for the C-terminal prenyl-modification of Ras, which is obligatory for membrane anchorage and proper functioning^54^. This may explain the weak cytoplasmic redistribution of H-ras at low amino acid levels (Supplementary Fig. 2A) and upon SREBP1 knockdown (Supplementary Fig. 4E) as a consequence of a decreased mevalonate pathway activity.

Importantly, our data suggest that the opposite regulation of the two Ras isoforms may be relevant in the stem cell or cancer stem cell context. Oncogenic K-ras more potently increased stemness properties than H-ras (Supplementary Fig. 5B). Moreover, the feedback from mTORC1 activity to Ras/MAPK-output was effectively reprogrammed, if oncogenic K-ras was expressed (compare Supplementary Figs 3 and 3D). Previous works by the McCormick and Settleman groups suggest that K-ras drives self-renewal and stemness properties^25, 26^. Our data here now suggest that in nutrient deprived niches with little growth factor support, suppressed mTORC1 activity may in fact strongly promote stemness in oncogenic K-ras transformed cells. In agreement with this, hypoxia, which also represses mTORC1, is known to support niches of quiescent cancer stem cells^55–57^. By contrast, under nutrient replete, normal growth conditions stemness is more moderately promoted (Fig. 3A,C) by the much weaker stemness driver H-ras (Supplementary Fig. 5B)^26^. We therefore tentatively propose that the known (patho)physiological effects of nutrient starvation or mTORC1 pathway inhibition are tightly linked to this Ras-isoform specific feedback. Consequently, we suggest that this coupling of two major tumorigenic pathways (PI3K/mTORC1 and Ras/MAPK) needs to be taken into account, when devising mTORC1-pathway targeted therapies. However, with a partial inhibition of mTORC1-activity in K-ras transformed cells one would expect a similar, stemness-enhancing outcome as we observe here with lowered amino acid levels. In these cases, it may be necessary to additionally block specifically K-ras, which has anyways advanced as the primary target amongst the Ras isoforms^18^. However, a number of drug targeting approaches that aim for inhibiting Ras activity with direct binders to the highly homologous G-domain or any other common regulator of Ras activity (such as GEFs)^58^, are very likely to hit all Ras isoforms fairly indiscriminately. With the advancement of anti-Ras tool compounds, we will hopefully not only see their anti-mitotic and pro-apoptotic effects on the primary tumour. Instead, data which document their potential to repress the aggressiveness of tumours as reflected by the tendency to metastasise and relapse, which are both tightly linked to cancer cells stemness^59, 60^, may be more relevant to predict success in the clinic.

## Materials and Methods

### Materials

Reagents were purchased from the following providers. Antibodies directed against Akt (no. 9272), phospho-T202/Y204 Erk (catalogue no. 9101), Erk (no. 9102), phospho-T389 S6K1 (no. 9234), S6K1 (no. 9202), phospho-S235/S236 S6 (no. 2211), S6 (no. 2217) and Raptor (no. 2280) came from Cell Signaling Technology (Danvers, MA, USA); phospho-T308 Akt (no. MAB7419) from R&D Systems (Wiesbaden, Germany); galectin-1 (no. 500-P210) from Peprotech (Hamburg, Germany); SREBP1 (no. sc-8984), and secondary anti-mouse (no. sc-2954) and anti-rabbit (no. sc-2004) were from Santa Cruz (Paso Robles, CA, USA); anti-GFP (no. 3999–100) was obtained from Bio-Vision (Milpitas, CA, USA) and β-actin (no. A1978) from Sigma (Sigma-Aldrich, Helsinki, Finland). Anisomycin and diacetoxyscirpenol came from Tocris Bioscience (Bristol, UK), harringtonine was from Santa Cruz, cycloheximide was from Acros Organics (Thermo Fisher Scientific), emetine 2HCL was from Calbiochem (Espoo, Finland), bortezomib was purchased from Cell Signaling Technology, compactin, propranolol and MG132 were from Sigma. Basal Medium Eagle (BME), 50x amino acids solution, Opti-MEM, horse serum, 50x B27 supplement, as well as transfection reagents RNAiMAX and Lipofectamine 3000 came from Thermo Fisher Scientific; Polyplus (Illkrich-Graffenstaden, France) was the provider for the transfection reagent JetPrime; Roswell Park Memorial Institute (RPMI), Dulbecco’s Modified Eagle Medium (DMEM), FGF and EGF came from Sigma.

### DNA constructs and siRNA

Plasmids pmGFP-RasG12V, pmCherry-RasG12V and pcDNA3-Gal-1 were described before^27, 53, 61, 62^. pcDNA3.1-2xFLAG-SREBP-1c was a gift from Timothy Osborne (Addgene plasmid #26802)^63^. pRK7-HA-S6K1-F5A-E389-R3A was a gift from John Blenis (Addgene plasmid #8991)^64^. mRFP-PASS was a gift from Guangwei Du^45^. The human gene directed siRNAs used in this study were bought from Dharmacon as ON-TARGET plus siRNA pools or from Qiagen as Flexi-Tube Gene Solution siRNA: scrambled siRNA (D-001810-10-20) and SREBP1 (L-006891-00) were from Dharmacon; siRNA directed against S6K1 (GS6198) and Raptor (GS57521) were from Qiagen. JetPrime was used for siRNA transfection unless otherwise stated.

### Cell culture

Human Embryonic Kidney 293-EBNA (HEK), Hs578T and MDA-MB-231 cells were cultured and transfected following exactly the same protocol described in ref. 65.

### FRET sample preparation

#### Compound treatments

HEK cells were plated on a 6-well plate with glass coverslips and thereafter transfected with donor-construct DNA (mGFP-tagged Ras constructs) for control samples, or together with acceptor-construct DNA (DNA ratio 1:3) as previously described^65^. After a day of expression, they were then treated with either control (DMSO 0.1% (v/v)), 0.4 μM bortezomib, 100 μM of propranolol or 2 μM of either of the protein synthesis inhibitors. 0.1% was the maximal final DMSO concentration.

To assess amino acid increase effect on Ras nanoclustering, 24 h after transfection cells were switched to either BME media (1x), which contains physiological concentrations of amino acids (low amino acid), or BME media supplemented with amino acids to a final concentration that is fourfold (4x) or eightfold (8x) higher, in the absence or presence of serum. After 12 h of incubation with the compounds, 4% PFA/PBS for 15 min was used for cell fixation. After a PBS-wash, samples were mounted with Mowiol 4–88 (Sigma-Aldrich). To assess phosphatidic acid (PA) effect on Ras nanoclustering, PA was suspended in chloroform, dried by vacuum centrifugation and resuspended at 10 mM concentration in 150 mM NaCl and 10 mM Tris·HCl pH 8, and it was immediately added to culture dishes at 100 μM final concentration and incubated for 45 min^66^ prior cell fixation.

#### Protein overexpression/knockdown

For SREBP1 protein overexpression or protein knockdowns, the cells were cotransfected with mGFP-/and mCherry-Ras FRET pairs (DNA ratio 1:3) or with mGFP-Ras/mRFP-PASS FRET pairs (DNA ratio 1:3) for co-clustering FRET experiments, following the instructions previously described^65^, together with either 1.5 μg empty vector (control) or plasmid encoding the mature form of SREBP1 (mSREBP1c) for its overexpression, or with either scrambled siRNA (control), siRNA directed against S6K1 (60 nM), Raptor (80 nM) or SREBP1 (25 nM) in the case of protein knockdown.

### FRET-imaging using fluorescence lifetime microscopy (FLIM)

The fluorescence lifetime of mGFP was determined using a fluorescence lifetime imaging attachment (Lambert Instruments, Groningen, Netherlands) on an inverted microscope (Zeiss AXIO Ovserver.D1, Jena, Germany) as previously described^27, 33^. For each condition, the fluorescence lifetime was measured typically for >30 cells from three independent biological repeats. From the obtained fluorescence lifetimes the apparent FRET efficiency was calculated as described in ref. 33.

### Immunoblotting

Western blotting was performed as described previously^36^. Membranes were immunoblotted with the mentioned antibodies and scanned using a ChemiDoc MP system (Bio-Rad, Hercules, CA, USA). Image-Lab software (Bio-Rad) was used for densitometric analysis.

### Sphere formation assay

HEK wildtype transiently expressing mGFP-H-rasG12V or mGFP-K-rasG12V, or MDA-MB-231 (K-rasG13D) and Hs578T (H-rasG12D) cells were used for sphere formation assays. For experiments with protein expression and knockdown, cells were cultured and transfected according to the protocol followed in ref. 65. For amino acid treatment experiments, cells were first subcultured twice in BME media at 1x, 4x or 8x amino acid concentration, in the presence of serum. Cells were then transferred to 48-well suspension culture plates and grown for 9 days in BME media with the same amino acid concentration and supplemented with B27, EGF and FGF. The spheres were analysed, normalized and statistically compared as previously described^27, 36, 65^.

### Determination of intracellular amino acid levels

Cells were serum starved for 16 h and treated for 1 h with the specified compounds in 96-well plates. Amino acid levels were quantified in triplicate using an L-Amino Acid Assay Kit (Abcam) according to the manufacturer’s instructions. Briefly, cells were washed with PBS and lysed in an assay buffer. A standard curve for quantification was established, using L-amino acid standards in a concentration range from 0 to 80 nmol/ml. Fifty microlitres of the reaction mix was added to each well containing the L-amino acid standard or test samples and the reaction was incubated for 30 min at 37 °C. The L-amino acid levels were quantified using fluorometric analysis on a BioTek Synergy 2 multi-mode microplate reader (BioTek Instruments, Winooski, VT, USA) at excitation and emission wavelengths of 535 nm and 590 nm, respectively, and were normalized to cell numbers in parallel wells.

### Confocal Imaging

Cells for sample preparation were grown on glass bottom 10 mm microwell dishes (MatTek corporation, Ashland, MA, USA). Living HEK cells were imaged using a spinning disk confocal microscope as described in ref. 65. Images were treated with ImageJ 1.49n (National Institutes of Health, Bethesda, MD, USA) to adjust for contrast and to display them on a gray-scale.

### Analysis of CD44^+^/CD24^**−**^ cells by flow cytometry

Wildtype HEK cells or HEK that were transfected with mGFP-H-rasG12V or mGFP-K-rasG12V, were cultured for 9 days as spheres with indicated SREBP1 manipulations. They were then analyzed using the flow cytometric protocol and analysis steps adapted from ref. 27. Briefly, a dot-plot with four quadrants for, CD24+ controls, CD44+ controls, double-stained cells, as well as non-stained control cells was established. Then the percentage change of CD44+/CD24− cells was determined from differences between non-treated controls and samples.

### Statistical analysis

The statistical significance of comparisons in Western blotting was evaluated with Student´s t-test. In the remainder of experiments statistical significance was determined by analysis of variance (ANOVA) together with Tukey’s honest significance difference test (Tukey’s HSD) in GraphPad PRISM software. The level of statistical significance is indicated as ns, not significant; *p < 0.05; **p < 0.01; ***p < 0.001; ****p < 0.0001.

## Electronic supplementary material

Supplementary Information
