# Supplementary material for: Opposite feedback from mTORC1 to H-ras and K-ras4B downstream of SREBP1
Source: Sci Rep. 2017 Aug 21;7:8944. doi: 10.1038/s41598-017-09387-8 (PMC5567141; doi:10.1038/s41598-017-09387-8)
Supplement: Supplementary file 1 — Supplementary Information [file 41598_2017_9387_MOESM1_ESM.pdf]

Supplementary information

Opposite feedback from mTORC1 to H-ras and K-ras4B downstream of SREBP1

Authors

Itziar MD Posada[1,2], Benoit Lectez[1,2], Farid A Siddiqui[1], Christina Oetken-Lindholm[1], Mukund Sharma[1], Daniel Abankwa[1,*]

[1] Turku Centre for Biotechnology, Åbo Akademi University, Tykistökatu 6B, 20520 Turku, Finland

[2] Co-first author

* Correspondence: daniel.abankwa@utu.fi; tel. +358 2 333 6969

Supplementary Figures

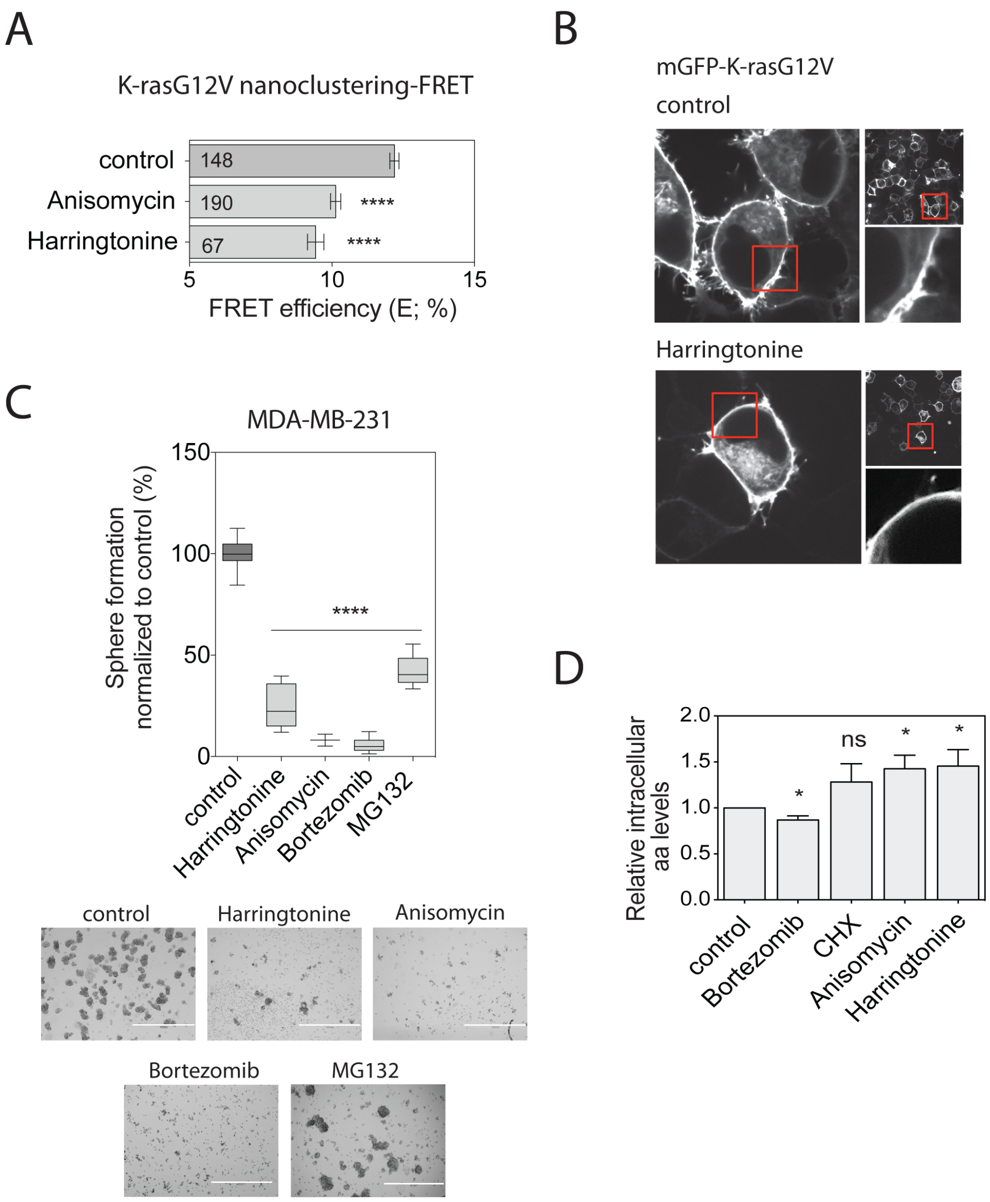

Supplementary Figure 1.

(A) Nanoclustering-FRET analysis in HEK cells coexpressing mGFP- and mCherry-tagged K-rasG12V. Cells were treated for 24 h with either DMSO control, 2 μM anisomycin or 2 μM harringtonine. The numbers in the bars indicate the number of analyzed cells (mean ± SEM, n≥3).

(B) Confocal images of mGFP-K-rasG12V transiently expressed in HEK cells treated

with DMSO control (*left*) or 2 μM harringtonine (*right*). Representative images from two independent experiments are shown.

(C) Mammosphere-forming efficiency of MDA-MB-231 cells grown in non-adherent conditions. Mammospheres were allowed to form for 6 days and treated for additional 3 days with DMSO control, 2 μM of harringtonine, 2 μM of anisomycin, 0.4 μM of bortezomib or 0.5 μM of MG132. On the top, the mammosphere-forming efficiency was measured as the number of spheres formed and normalized to control (mean ± SEM, n=2). On the bottom, representative images of mammospheres are shown as indicated. Scale bar represents 1000 μm.

(D) Determination of intracellular amino acid levels. BHK cells were serum starved for 16 h and subsequently treated for 1 h with DMSO control, 0.4 μM bortezomib, 0.18 μM CHX, 2 μM anisomycin or 2 μM harringtonine. Note that CHX was used at a minimal concentration here that reportedly does not fully block protein synthesis [1,2]. Amino acid levels are shown as mean ± SEM of triplicate samples relative to control. Statistical significance levels are annotated as ns, not significant; *, $p < 0.05$; ****, $p < 0.0001$.

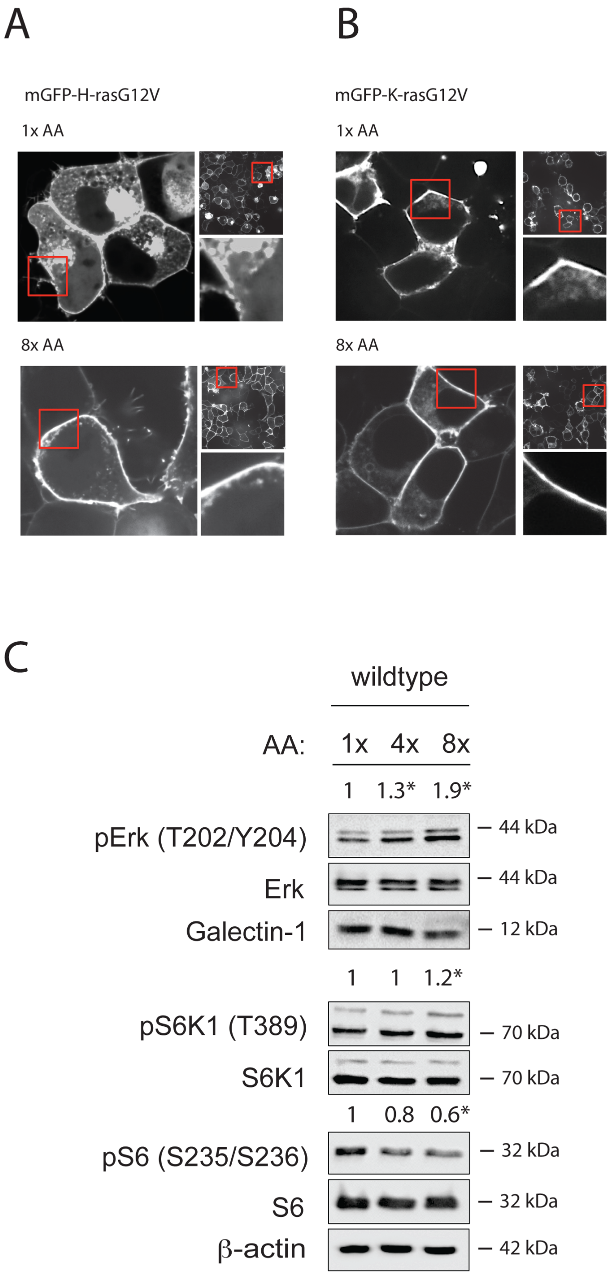

Supplementary Figure 2.

(A-B) Confocal images of mGFP-H-rasG12V **(A)** or mGFP-K-rasG12V **(B)** expressed in HEK cells incubated for 24 h in BME medium complemented with serum and 1x (*top*) or 8x (*bottom*) amino acid concentration. Representative images from two independent experiments are shown.

(C) Western blot analysis of Ras and mTORC1 signalling in wildtype HEK cells. Cells were treated as in **(A-B)** under serum-starved conditions. Numbers indicate the

ratio of phosphorylated/ cleaved protein normalized to total protein/ actin levels, respectively (n=4). Statistical significance levels are annotated as *, $p < 0.05$.

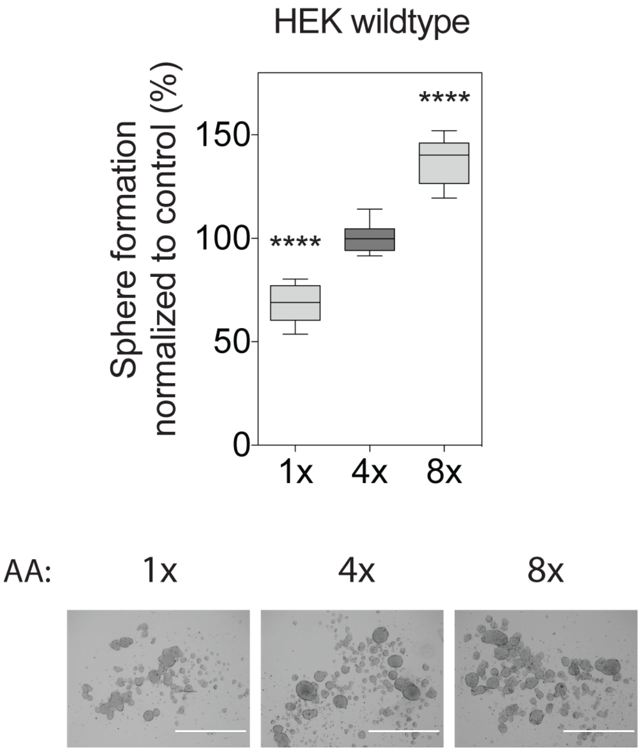

Supplementary Figure 3.

Sphere-forming efficiency of wildtype HEK cells grown under non-adherent conditions. Spheres were allowed to grow with increasing aminoacid concentrations for 9 days. On the left, the sphere-forming efficiency was measured as the number of spheres formed and normalized to control (n=3). On the right, representative images of spheres are shown as indicated. Scale bar represents 1000 μm. Statistical significance levels are annotated as ****, $p < 0.0001$.

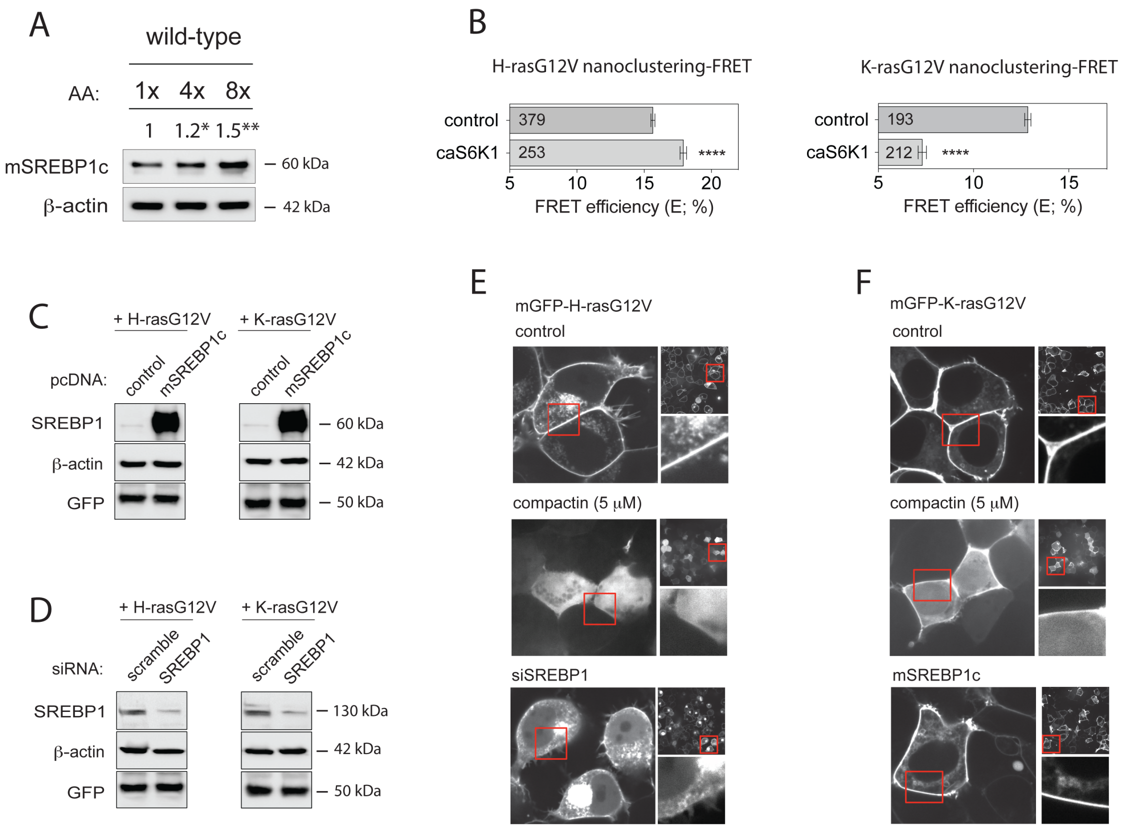

Supplementary Figure 4.

(A) Western blot analysis of mSREBP1c in wildtype HEK cells. Cells were incubated for 24 h with increasing concentrations of amino acids (1x, 4x or 8x) in the absence of serum. Numbers indicate β-actin normalized mSREBP1 levels (n=3).

(B) Nanoclustering-FRET analysis in HEK cells transfected with constitutively active form of S6K1, caS6K1, or empty vector, co-expressing mGFP- and mCherry-tagged H-rasG12V (*left*) or K-rasG12V (*right*). The numbers in the bars indicate the number of analyzed cells (mean ± SEM, n=4).

(C-D) Western blot analysis of SREBP1 mature form expression, mSREBP1c **(C)**, and of SREBP1 knockdown **(D)** in HEK cells expressing mGFP-H-rasG12V (*left*) or mGFP-K-rasG12V (*right*).

(E-F) Confocal images of mGFP-H-rasG12V **(E)** or mGFP-K-rasG12V **(F)**

expressed in HEK cells, and treated with compactin, a mevalonate pathway inhibitor, or upon SREBP1 knockdown **(E)** or mSREBP1c overexpression (**F**). Representative images from two independent experiments are shown.

Statistical significance levels are annotated as *, $p < 0.05$; **, $p < 0.01$; ****, $p < 0.0001$.

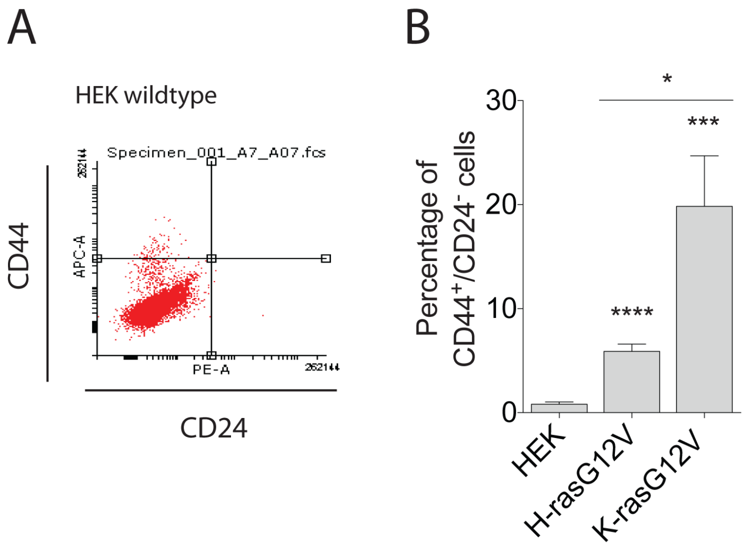

Supplementary Figure 5.

(A) CD44/CD24 FACS profiles are shown for wildtype HEK cells.

(B) Shown is the average percentage of $CD44^{+}/CD24^{-}$ for wildtype HEK cells and HEK cells expressing H-rasG12V or K-rasG12V. Error bars denote the SEM from three independent experiments performed in duplicate. Statistical significance levels are annotated as *, $p < 0.05$; ***, $p < 0.001$; ****, $p < 0.0001$.

Supplementary References

1. Ahearn, I. M. *et al.* FKBP12 Binds to Acylated H-Ras and Promotes Depalmitoylation. *Mol. Cell* **41,** 173–185 (2011).
2. Najumudeen, A. K. *et al.* Phenotypic Screening Identifies Protein Synthesis Inhibitors as H-Ras-Nanocluster-Increasing Tumor Growth Inducers.

Biochemistry **54,** 7212–7221 (2015).
